# C/EBPα Regulates *PxTreh*1 and *PxTreh*2 Trehalase-Related Bt Resistance in *Plutella xylostella* (L.)

**DOI:** 10.3390/insects13040340

**Published:** 2022-03-30

**Authors:** Jia Liu, Zheming Liu, Haihao Ma, Yaying Yu, Chengjia Zhang, Wei Zheng, Yilong Man, Hang Zhu, Yong Zhou, Xi Chen, Xiaomao Zhou, Aiping Zeng

**Affiliations:** 1 College of Plant Protection, Hunan Agricultural University, Changsha 410128, China; jialiuv@hunaas.cn; 2 Institute of Agricultural Biotechnology, Hunan Academy of Agricultural Sciences, Changsha 410125, China; liuzheming2007@hunaas.cn (Z.L.); mhh8486@hunaas.cn (H.M.); zhangchengjia@hunaas.cn (C.Z.); zhengweiaimm@hnu.edu.cn (W.Z.); yilongman@hunaas.cn (Y.M.); zhumstrong@hunaas.cn (H.Z.); qmbchg@hunaas.cn (Y.Z.); xichen@hunaas.cn (X.C.); 3 Hunan Rice Research Institute, Changsha 410125, China; yuyaying@hunaas.cn

**Keywords:** *P. xylostella*, *PxTreh*1, *PxTreh*2, C/EBPα, expression regulation

## Abstract

**Simple Summary:**

The diamondback moth (*Plutella xylostella*) is a major agricultural pest of cruciferous vegetables and crops worldwide, causing economic losses of up to USD 5 billion annually. The long-term use of insecticides leads to the rapid evolution of resistance in insects, which greatly increases the difficulty of controlling pests. Trehalase regulates energy metabolism in insects by converting trehalose into two glucose molecules. The existence of trehalase is critical for insect flight and larval stress resistance. However, whether trehalase participates in the development of pesticide resistance remains unclear. In this study, we found that the activity of trehalase and the levels of gene expression in Bt-resistant and field populations of *P. xylostella* were significantly higher than they were in the susceptible strains. By analyzing the promoter sequences of *PxTreh*1 and *PxTreh*2, we confirmed the interaction between C/EBPα and the *PxTreh*2 promoter. The findings of this study suggest that C/EBPα mediates the adaptability of *P. xylostella* to adverse environmental stressors by regulating the expression of trehalase.

**Abstract:**

Trehalase regulates energy metabolism in insects by converting trehalose into two glucose molecules. High amounts of trehalase are critical for insect flight and larval stress resistance. However, whether trehalase participates in the development of pesticide resistance remains unclear. In this study, we explored this phenomenon and the mechanism that underlies the regulation of *Trehalase* transcription. We found that overexpression of *PxTreh*1 and *PxTreh*2 induced *Bacillus thuringiensis* (Bt) resistance in *Plutella xylostella*. The promoter sequences of *PxTreh*1 and *PxTreh*2 were also cloned and identified. The dual-luciferase reporter system and RNA interference technology revealed that the expression of *PxTreh*1 and *PxTreh*2 genes is possibly regulated by the CCAAT enhancer-binding protein (C/EBPα). A yeast one-hybrid experiment confirmed the interaction between C/EBPα and the *PxTreh*2 promoter. The findings of this study suggest that C/EBPα mediates the adaptability of *P. xylostella* to adverse environmental stressors by regulating the expression of trehalase.

## 1. Introduction

Trehalase is an enzyme present in numerous bacteria, fungi, plants, and invertebrates. The enzyme regulates energy metabolism and plays a critical role in the growth, development, molting, and metamorphosis of hemolymph insects. Given these functions, trehalose is also called the "blood sugar" of insects [1,2,3]. Trehalase catalyzes the hydrolysis of one trehalose molecule into two glucose molecules. The energy generated drives numerous physiological and biological activities [4,5,6], including flying [7]. The first trehalase identified was a soluble trehalase, cloned from *Tenebrio molitor*. It exists freely in the cytoplasm and degrades endogenous trehalose, primarily in the circulatory and digestive systems, such as the hemolymph, midgut, and ovaries [8]. In 2005, a membrane-bound trehalase gene from *Bombyx mori* was isolated. It was designated TRE2 and was found to be an extracellular enzyme, primarily found in the microvilli or basolateral membranes and bound to mitochondria in the muscle. The enzyme catalyzes the uptake and assimilation of exogenous trehalose and is highly expressed in the fat body, midgut, and malleoli [1,9,10,11,12,13,14]. Inhibition or disrupted expression of TRE disrupts molting, reduces chitin synthesis, impairs proper flying, and causes malformation of the wings, weight loss, stunted growth, and even death [3,15].

CCAAT enhancer-binding protein (C/EBPα), an important transcription factor, regulates gene transcription, growth, cycle, the differentiation of cells, immune response, energy metabolism, tumorigenesis, and apoptosis [16,17]. C/EBPα regulates energy metabolism [18]. Mice models revealed that a deficiency in C/EBPα causes death after birth because C/EBP is required for macrophage activation and maintenance of energy metabolism in the skeletal muscles [19]. C/EBPα is also a key transcription factor that regulates myeloid differentiation and modulates the expression of C/EBPα, causing resistance to imatinib, and is essential for chronic granulocytic leukemia treatment [20].

Insects develop resistance to various stressors, such as extreme temperatures and chemicals. For insect pests, resistance to pesticides has crop-production and economic significance [21]. The resistance of insects to insecticides requires abundant energy relative to their susceptible counterparts [22]. Fludioxonil-resistant *Aphis gossypii* [21,23], *Bacillus thuringiensis* (Bt)-resistant *Bactrocera dorsalis* [24], trifloxymethoxazole-resistant *Frankliniella occidentalis* [25], indoxacarb-resistant *Helicoverpa armigera* [26], and phosphine-resistant *Tribolium castaneum*, *Rhyzopertha dominica*, and *Oryzaephilus surinamensis* all display the costs of resistance, including reduced fecundity, longer developmental time, shorter adult life span and reproductive period, and reduced tolerance to cold temperatures [27]. Interestingly, if resistant insects can store sufficient energy material, the cost of resistance can be offset. The pyrethroid-resistant maize weevil *Sitophilus zeamais* does not exhibit the cost of resistance because of its increased energy storage, respiration rate, and response to insecticides by secreting detoxifying enzymes [28]. This finding underlines the need to clarify the role of trehalase in stress and environmental-pressure responses in insects, and the molecular mechanism underlying this process. We cloned the diamondback moth (*Plutella xylostella*) trehalase promoter into the pGL3-Basic vector. The expression of *P. xylostella* trehalase in the field in the insecticide-resistant and the Bt-sensitive *P. xylostella* populations was then compared. The regulation of trehalase expression was also analyzed. Finally, the role of *P. xylostella* trehalase in insects under different stressors was also clarified.

## 2. Materials and Methods

### 2.1. Insects and Cell Line

Field *P. xylostella* specimens were collected from Hanshou County (HS), Hunan Province, China (111.975° E, 28.957° N) and reared in an insectarium at the Hunan Agricultural Biotechnology Institute (Changsha, China) at 25 ± 1 °C, 65% ± 5% relative humidity, and with a light cycle of 16 h light:8 h dark. The larvae were fed cabbage leaves, whereas the adults were fed a 5% solution of honey in water. RNA for cloning the *Τreh* gene was extracted on the second day of the fourth-instar larvae. Analyses of the profile of gene expression were performed using fourth-instar larvae and adults. The Bt-susceptible (Bt-S) and Bt-resistant (Bt-R) *P. xylostella* strains were obtained from the Institute of Vegetables and Flowers, Chinese Academy of Agricultural Sciences (Beijing, China).

*Trichoplusia ni* Hi5 cells for the dual-luciferase reporter assay were cultured at 27 °C in TNM-FH insect medium (Sigma-Aldrich, St. Louis, MO, USA) supplemented with 10% fetal bovine serum (Gibco BRL, Grand Island, NY, USA).

### 2.2. Trehalase Activity Assay

3,5-Dinitrosalicylic acid colorimetry was used to measure the content of the reduced sugar (glucose) produced by trehalase (THL) catalysis. One gram of tissue will convert trehalose to 1 μg glucose per min in the reaction system, which is defined as 1 U of enzymic activity. Five fourth-instar larvae from three *P. xylostella* populations (Bt-S, Bt-R, and HS) were used for the trehalase activity assay. Each sample consisted of five one-day-old fourth-instar larvae. The enzymatic activity of THL was measured using a THL kit (Solarbio Science & Technology Co., Ltd., Beijing, China) following the manufacturer’s instructions.

### 2.3. Quantitative Real-Time PCR

Tissue-specific gene expression analysis was performed using three larval and adult tissues of *P. xylostella* [29]. Total RNA was extracted from different samples using RNA Isolater Total RNA Extraction Reagent following the manufacturer’s instructions (Vazyme, Nanjing, China). The extracted RNA was quantified using a NanoDrop™ 1000 (Thermo Fisher Scientific, Waltham, MA, USA). cDNA was synthesized using 1 µg of RNA sample and HiScript Q RT SuperMix (+gDNA wiper). The total reaction volume was 20 µL. The synthesized cDNAs were stored at −80 °C until further use. Quantitative analyses of *P. xylostella* cDNA samples at different life stages and treated with RNA interference technology (RNAi) were performed using a FastStart Essential DNA Green Master Kit (Roche, Basel, Switzerland). The cDNA sample (15 ng) was reverse transcribed from the total RNA and used as the PCR template. The *PxTreh* genes were amplified using specific primers listed in Table 1. Ribosomal protein S4 (rpS4, XM_011555372) of *P. xylostella* was amplified and used as the internal control for the quantitative PCR analysis at different insect growth stages [30,31]. Elongation factor 1 (EF1, EF417849) was also amplified and used to validate the quantitative results (primers shown in Table 1). A LightCycler^®^ 96 PCR instrument (Roche Molecular Systems, Inc., Basel, Switzerland) was used for the analysis of gene expression. The two-step amplification process was performed under initial denaturation at 95 °C for 30 s, subsequent denaturation at 95 °C for 10 s, and annealing at 59 °C for 30 s. The process was performed through 40 cycles. The relative expression of the gene was calculated using the 2^−∆∆CT^ method [32,33].

### 2.4. 5’ RNA Ligase-Mediated Rapid Amplification of cDNA Ends (5’ RLM-RACE)

The *PxTreh*1 (XM_038109193) and *PxTreh*2 (XM_038109919.1) genes were amplified using specific primers based on the cDNA synthesized from *P. xylostella* RNA. The transcription start site of the *PxTreh* gene was determined using a First Choice RLM-RACE Kit (Invitrogen, Carlsbad, CA, USA). Briefly, total RNA from one-day-old third-instar *P. xylostella* larvae was extracted using RNA Isolater Total RNA Extraction Reagent (Vazyme Co., Ltd., Nanjing, China) following the manufacturer’s instructions and treated with calf intestinal phosphatase (CIP) to remove phosphates at the 5′ end of the degraded mRNA and structural RNA. The RNA was then purified and treated with tobacco acid pyrophosphase (TAP) to remove the 5′-7-methylguanine cap of the full-length mRNA, leaving the 5’-monophosphate section. A 45-base RNA adapter oligonucleotide was ligated to the capless mRNA using T4 RNA ligase. The cDNA was synthesized using a random-primed reverse transcription reaction. The full-length 5′ untranslated region of the *PxTreh* mRNA was amplified using nested PCR. Two sense primers corresponding to the RNA adapter were purchased alongside the kit, and the antisense primers (outer and inner primers, Table 1) were specific to the *PxTreh* mRNA. The first PCR was performed using the 5’ RACE anchor primer and the *PxTreh* outer primer (Table 1). The second nested PCR was performed using the 5’-RACE and the *PxTreh* inner primers (Table 1). The amplification conditions included initial denaturation at 95 °C for 3 min, subsequent denaturation through 30 cycles at 95 °C for 15 s, annealing at 60 °C for 15 s, elongation at 72 °C for 45 s, and a final extension at 72 °C for 5 min. The final product was purified using a Fast Pure Gel DNA Extraction Mini Kit (Vazyme) before cloning into the pCE-Zero vector (Vazyme). The 5′ untranslated *PxTreh* region was confirmed using Sanger sequencing.

### 2.5. Cloning of the Promoter and TFs

The RACE sequences were searched in the *P. xylostella* genome database. Two DNA fragments were obtained: scaffold NW_011952223.1 and NW_011952162.1. The primers were designed based on the gene sequences (Table 1). The truncated promoter fragment was inserted into the pGL3-Basic vector by seamless cloning.

Potential CREs for TF binding in the *PxTreh* promoter were predicted using the JASPAR database (http://jaspar.genereg.net (accessed on 9 March 2021)). C/EBPα was predicted to bind both *PxTreh* promoters. The coding sequences (CDSs) of C/EBPαin *P. xylostella* were extracted from the GenBank database (https://www.ncbi.nlm.nih.gov/ (accessed on 24 March 2021)) under accession numbers LOC105380784. A sequence analysis showed the encoded amino acid sequence was consistent with the reference sequence. The full-length CDSs were amplified using corresponding specific primers (Table 1). The reaction conditions included pre-denaturation at 95 °C for 3 min, subsequent denaturation through 35 cycles at 95 °C for 15 s, annealing at 60 °C for 15 s, elongation at 72 °C for 30 s, and a final extension at 72 °C for 5 min.

### 2.6. PxTreh Promoter-Reporter Constructs

The function of the putative promoter was determined by linking each promoter sequence to a firefly luciferase reporter gene sequence. For the *Pxtreh*1 promoter, the promoter–luciferase constructs contained 731 (−329–402), 828 (−426–402), 889 (−487–402), 947 (−545–402), 1012 (−610–402), and 1590 (−1188–402) bases. For the *Pxtreh*2 promoter, the promoter–luciferase constructs contained 286 (−90–196), 337 (−141–196), 399 (−203–196), 450 (−254–196), 511 (−315–196), 1121 (−925–196), 1648 (−1452–196), and 2088 (−1892–196) bases. Each promoter region in the pGL3-Basic plasmid was amplified using PCR, and the PCR product was purified using agarose gel electrophoresis and a Fast Pure Gel DNA Extraction Mini Kit. The PCR product was transformed into *E. coli* strain DH5α, and the positive clones were selected and confirmed by sequencing.

### 2.7. Dual-Luciferase Activity Assay

*Trichoplusia ni* Hi5 cells were cultured at 27 °C for 24 h in 24-well plates that contained 500 μL of TNM-FH medium (Nest, Wuxi, Jiangsu, China) at a density of 1.5 × 10^5^ cells/well before transfection. The transfection was performed using a FuGENE^®^ HD Transfection Reagent (Promega, Madison, WI, USA) according to the manufacturer’s instructions. Briefly, 2.5 ng pRL-OpIE2 [34] and 0.6 μg recombinant plasmid construct, which contained the full-length promoter and truncated pGL3 sequence, were mixed with Transfection Reagent in 100 μL Grace (Gibco BRL), vortexed briefly, and stored at room temperature for 25 min. The DNA-lipid mixture was added to the cells dropwise, which were then cultured at 28 °C for 4 h before the transfection mixture was replaced with 500 μL TNM-FH medium and further cultured at 28 °C for 48 h for the dual-luciferase activity assay. To detect the activation of the promoter by transcription factor C/EBPα, 0.3 μg pGL3-*Pxtreh*1-P (−487 to 402) or pGL3-*Pxtreh*2-P (−203 to 196), 0.3 μg pEGFP-N1-C/EBPα, and 1 ng pRL-OpIE2 were also co-transfected into cells that were then cultured for 48 h as described above. At 48 h post-transfection, the cells were collected to measure the firefly and Renilla luciferase activities using the Dual Luciferase Reporter Assay Kit (Promega) and a Synergy 2 multi-code microplate reader from BioTek (Winooski, VT, USA), respectively, according to manufacturer’s instructions. All the reporter assays were repeated three times (*n* = 3), and the expression of the reporter genes was expressed as a mean of the three experiments ± SEM.

### 2.8. Y1H Assay

The yeast one-hybrid (Y1H) assay was performed to explore the direct interaction between C/EBPα and the *PxTreh*2 using a Matchmaker Gold Yeast One-Hybrid System (Clontech, Mountain View, CA, USA). The bait plasmids pAbAi-CRE containing *PxTreh*2 (−203–196) were linearized with BstBI and integrated into Y1HGold yeast to generate the bait population. Successful transformants were selected using SD/-Ura media. The minimum AbA concentration that inhibited the normal growth of the bait population on SD/-Ura medium was 100 ng/mL. The prey plasmid containing C/EBPα was constructed by subcloning the CDS into the pGADT7 vector, which was then transformed into the bait population. The selection of the transformants was performed on SD/-Leu media supplemented with 100 ng/mL Aureobasidin A (AbA). The positive control was the Y1HGold strain transformed with pGADT7-p53 and pAbAi-p53 plasmids, and the negative control was the Y1HGold strain transformed with an empty pGADT7 and the pAbAi-CRE plasmid.

### 2.9. dsRNA Synthesis and RNAi

Primers with T7 promoter sequences were designed based on the conserved domain of C/EBPα (Table 1). The C/EBPα dsRNA (dsC/EBPα) was synthesized using a MEGAscript RNAi Kit (Ambion, Austin, TX, USA). The dsC/EBPα gene was injected into one-day-old *P. xylostella* fourth-instar larvae using microinjection tools (WPI, Sarasota, FL, USA). dsEGFP was used as the negative control [35]. Each larva was injected with 150 ng dsRNA, and a sample of three injected larvae was collected after 24 h for quantitative real-time PCR analysis.

### 2.10. Statistical Analysis

The differences between groups were analyzed using the Student’s *t*-test and a one-way analysis of variance (ANOVA). Tukey’s honestly significant difference test was used to analyze the differences between more than two groups. Data were analyzed using GraphPad Prism 9 (GraphPad Software, San Diego, CA, USA). The experiments were performed in triplicate, and the data were presented as the mean of the three experiments ± standard deviation (SD).

## 3. Results

### 3.1. Trehalase Activity Was Significantly High in Bt-R P. xylostella Larvae

**Figure 1:**
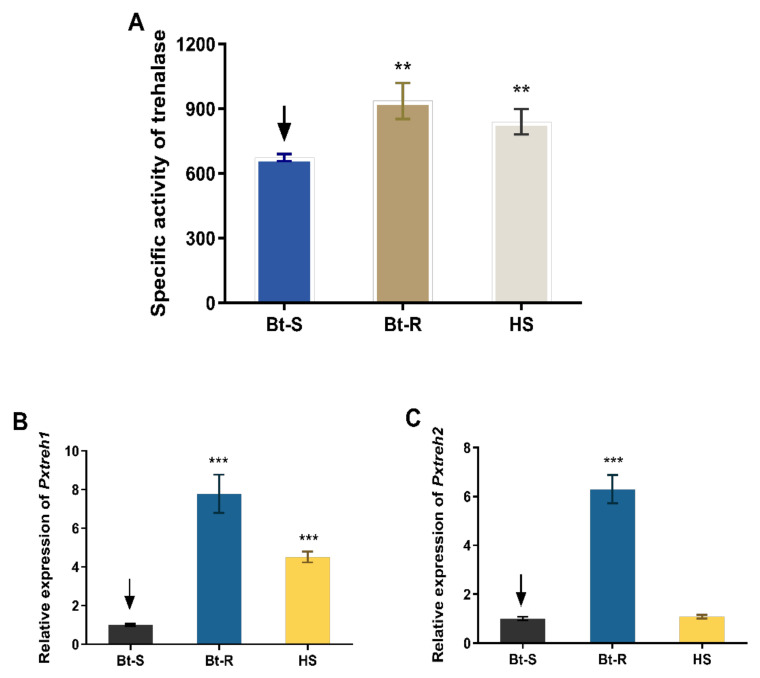
Trehalase activity and the relative expression of *PxTreh*1 and *PxTreh*2 genes in different *Plutella xylostella* larval populations. (**A**) The trehalase activity in different *P. xylostella* populations. (**B**) The relative *PxTreh*1 expression in different *P. xylostella* populations. (**C**) The relative *PxTreh*2 expression in different *P. xylostella* populations. Data are expressed as the mean of four independent experiments (*n* = 4). The mean of columns with different asterisks (*) are significantly different. ** *p* < 0.01. *** *p* < 0.001. The control groups in (**A**–**C**) are marked with a downward arrow.

Assays of enzyme activity revealed that the trehalase is highly active in the Hanshou (HS, 839.4 ± 59.3 U/g fresh weight), Bt-resistant (Bt-R, 936.1 ± 83.9 U/g fresh weight), and Bt-sensitive (Bt-S, 673.5 ± 16.4 U/g fresh weight) populations. In particular, the trehalase activities of Bt-R and HS were 1.39-fold and 1.24-fold, respectively, relative to that of the Bt-S group (Figure 1A).

### 3.2. Expression of PxTreh1 and PxTreh2 Genes Were Significantly High in the HS and Bt-R P. xylostella Population

The expression of *PxTreh*1 in Bt-R and HS groups was 6.786 ± 0.4940 (95% confidence interval 5.578 to 7.995) and 5.295 ± 0.2926 (95% confidence interval 4.579 to 6.011), respectively, compared with that of the Bt-S group (Figure 1B). Additionally, the expression of *PxTreh*2 was significantly higher in the Bt-R population. Specifically, it was 3.521 ± 0.1438 times that of the Bt-S group. However, there was no significant difference in *PxTreh*2 expression between the HS and the Bt-S group (*p* = 0.175) (Figure 1C). These findings suggest that the high trehalase activity in the Bt-R and HS populations was caused by the overexpression of *PxTreh*1 or *PxTreh*2 genes.

### 3.3. The Expression of PxTreh Gene in P. xylostella Developmental Stages

**Figure 2:**
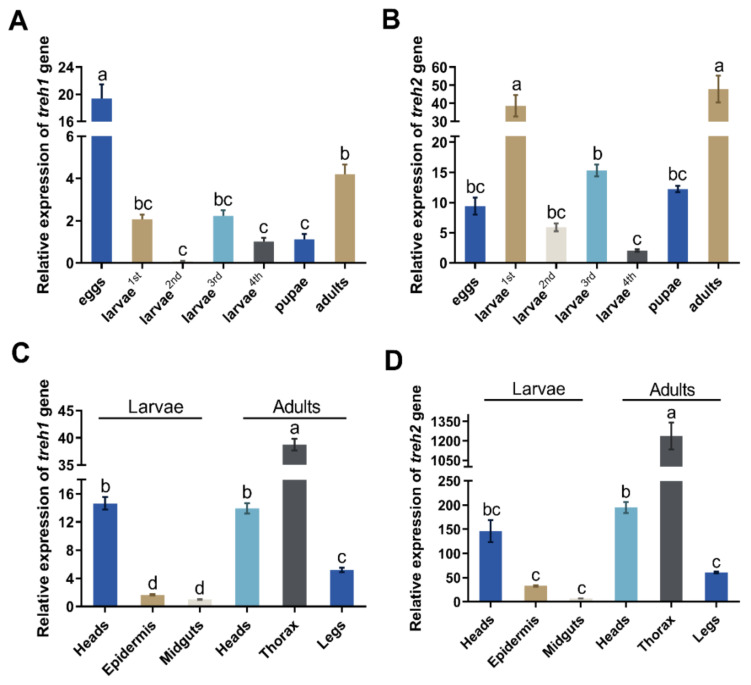
The relative expression (mean ± SE) of *Treh*1 and *Treh*2 in *Plutella xylostella*. (**A**) Expression of the *Pxtreh*1 genes in different development stages (first to fourth larvae, pupae, adults, and eggs). (**B**) The expression of *Pxtreh*2 genes in different developmental stages (first to fourth larvae, pupae, adults, and eggs). (**C**) The expression of *Pxtreh*1 in different tissues (the heads, epidermises, and midguts of the fourth-instar larvae and the heads, thoraxes, and legs of the adults). (**D**) The expression of *Pxtreh*2 in different tissues (the heads, epidermises, and midguts of the fourth-instar larvae and the heads, thoraxes, and legs of the adults). *Pxtreh*1 expression in the midguts of the fourth larvae was used as the control. Data are expressed as a mean of three independent experiments (*n* = 3). Endogenous EF1 was used as the internal control. Values with the same letter are significantly different at *p* < 0.05 (Tukey’s honest significance test). SE, standard error.

The *PxTreh*1 and *PxTreh*2 genes were expressed in all *P. xylostella* developmental stages (eggs, larvae, pupae, and adults). However, the expression of *PxTreh*2 was significantly higher than that of *PxTreh*1 in all developmental stages except the eggs, in which the *PxTreh*1 expression was the highest (Figure 2A,B). The expression of *PxTreh*2 was highest in the first-instar larvae and adults.

The expression of *PxTreh*2 was also higher than that of *PxTreh*1 in different tissues. The expression of *PxTreh*1 was higher in the thorax and lower in the midgut tissues. The expression of *PxTreh*2 displayed a similar pattern (Figure 2C,D).

### 3.4. Cloning of the PxTreh Promoter

**Figure 3:**
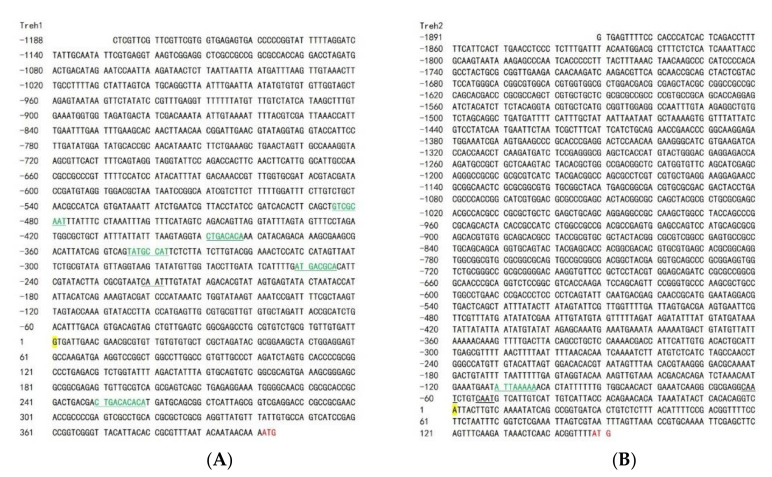
Analysis of the transcription start site of the cloned section. (**A**) Analysis of the *PxTreh*1 transcription start site. (**B**) Analysis of the *PxTreh*2 transcription start site. The inclined sequence was obtained using 5’ RLM-RACE. Yellow Nucleobase indicates the transcription initiation site, whereas the black underlined section is the *cis*-acting element CAAT box. The green and underlined sections are transcription factor binding sites. Skeleton alphabet sequence from RACE. RLM-RACE, RNA ligase-mediated rapid amplification of cDNA ends.

To identify the transcription initiation sites of the *PxTreh* gene, the 5′ end sequences of both cDNA of *PxTreh*1 (675 bp) and cDNA of *PxTreh*2 (445 bp) were first obtained using 5’ RLM-RACE (Figure 3). The sequences obtained by 5′ RACE were searched in the *P. xylostella* genome database, and two DNA fragments (scaffold NW_011952223.1 and NW_011952162.1) were identified. The promoter sequences of *PxTreh*1 (1188 bp) and *PxTreh*2 (1891 bp) were then obtained and cloned into pGL3-Basic vectors. Moreover, the five *cis*-acting elements in the *PxTreh*1 promoter (−252, −346, −391, −486 and +250), and one *cis*-acting element in the *PxTreh*2 promoter (−111) were predicted using the JASPAR database (Figure 3).

### 3.5. The Promoter Activity of the PxTreh Gene

**Figure 4:**
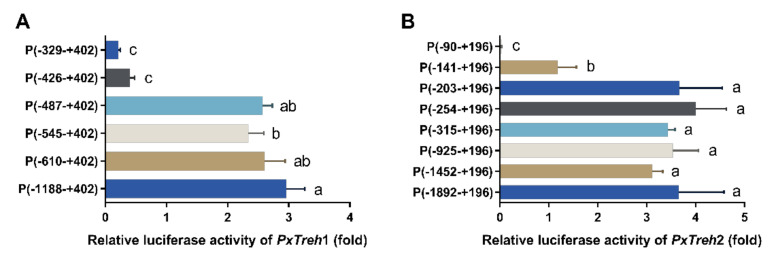
Detection of promoter activity by a dual-luciferase reporter assay. (**A**) The activity of progressive 5’ deletion of the recombinant *PxTreh*1 promoters between −1188 to −329. (**B**) The activity of the recombinant *PxTreh*2 promoters continuously cut from −1892 to −90. Values with the same letters are significantly different at *p* < 0.05 (Tukey’s honest significance test).

To accurately analyze the promoter-active region of *PxTreh*1, six pGL3-Basic recombinant plasmids that contained the *PxTreh*1 promoter, including P (−1188 to +402), P (−610 to + 402), P (−545 to +402), P (−487 to +402), P (−426 to +402), and P (−329 to +402), were constructed. The luciferase activity was measured by co-transfecting the pGL3 base vector and pRL-OpIE2 plasmids into the Hi5 cells. It was found that, compared with the promoter activity of P (−487 to +402), P (−426 to +402) reduced promoter activity by 84.3% (Figure 4A), suggesting that the region from −487 to +402 may be the main section that regulates promoter activity (Figure 4A).

Eight pGL3-Basic recombinant plasmid vectors based on *PxTreh*2, which included P (−1892 to +196), P (−1452 to +196), P (−925 to +196), P (−315 to +196), P (−254 to +196), P (−203 to +196), P (−141 to +196), and P (−90 to +196), were constructed. Truncating the promoters from −203 to −141 at the 5’ end significantly reduced the promoter activity of *PxTreh*1, and no transcription from the −90 to the +196 region was detected. These findings suggest that the elements responsible for *PxTreh*2 promoter activity are located within the −203 to +196 region (Figure 4B).

### 3.6. The C/EBPα Transcription Factor Enhances PxTreh Expression

**Figure 5:**
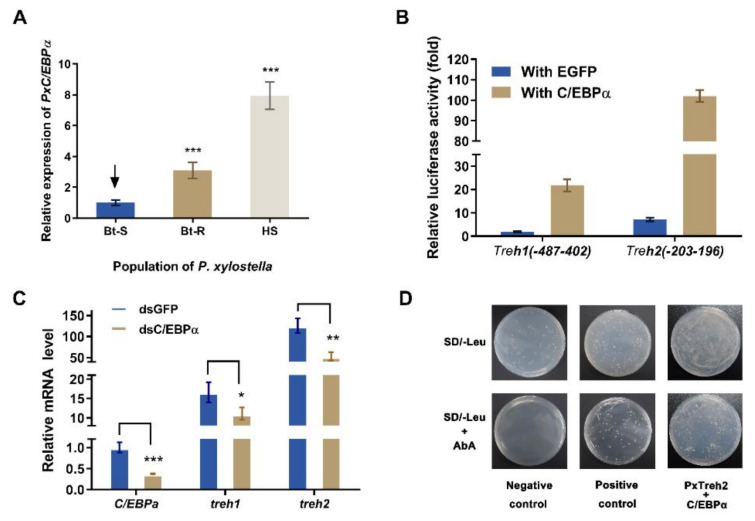
Effect of C/EBPα on the expression of *PxTreh* genes in vivo. (**A**) The expression of *PxC/EBP*α in the fourth-instar larvae of the Bt-S, Bt-R, and HS *Plutella xylostella*. The EF1 gene was used as an internal control. (**B**) Effects of C/EBPα on the activity of *PxTreh*1 and *PxTreh*2 promoters. The empty pEGFP-N1 vector that overexpressed green fluorescent protein (EGFP) was used as the control. (**C**) The relative expression of *PxC/EBP*α in larvae of the sensitive strain at 24 h post-injection with C/EBPα, dsGFP, or dsC/EBPα. The EF1 gene was used as the internal control. (**D**) The effect of C/EBPα on *PxTreh*2 promoter activity. The interaction between C/EBPα and the *PxTreh*2 promoter region was investigated using the Y1H assay. Bait vectors that contained the *PxTreh*2 promoter (−203 to +196) and a prey vector that contained C/EBPα were transferred into the Y1HGold yeast strain. The yeast was grown on SD/-Leu selective media with or without AbA. The data are presented as the average relative expression of genes in three experiments ± SEM. * *p* < 0.05. ** *p* < 0.01. *** *p* < 0.001. AbA, Aureobasidin A; SEM, standard error of the mean; Y1H, yeast one-hybrid. The control groups in (**A**) is marked with a downward arrow.

The transcription factors C/EBPα were predicted to regulate the expression of the *PxTreh* promoter region (Figure 3). To verify this, the expression of C/EBPα in different *P. xylostella* populations was measured. We found that the expression of C/EBPα in the Bt-R population was 2.06-fold and 6.87-fold in the HS population compared with the Bt-S group (Figure 5A). These findings suggest that C/EBPα regulates the expression of trehalase genes. In this study, a pEGFP-N1 vector that expressed C/EBPα was constructed. The plasmid was co-transfected with P (−487 to 402) of *PxTreh*1 and P (−203 to 196) of *PxTreh*2. P (−487 to 402) of the *PxTreh*1 co-expression of C/EBPα significantly increased by 10.60-fold compared with the co-expression of the EGFP group, and P (−203 to 196) of the *PxTreh*2 co-expression of C/EBPα was significantly increased by 13.21-fold compared with the co-expression of the EGFP group (Figure 5B). These findings showed that C/EBPα enhanced the activity of *PxTreh*1 and *PxTreh*2 promoter regions, indicating that C/EBPα participates in regulating the expression of *PxTreh* in *P. xylostella* larvae. 

An RNA interference (RNAi) of C/EBPα was performed to confirm the regulatory effect of C/EBPα on *PxTreh*1 and *PxTreh*2 expression in vivo. The expression of C/EBPα decreased significantly 24 h after the injection of ds *PxC/EBP*α RNA. Compared with the injection of GFP dsRNA, the RNAi of *PxC/EBP*α significantly decreased the expression of *PxTreh*1 and *PxTreh*2 (*p* < 0.01) by 33.05% and 57.76%, respectively (Figure 5C). 

### 3.7. C/EBPα Interacts with the PxTreh2 Promoter Region

A Y1H assay was further performed to explore the interaction between C/EBPα and the *PxTreh*2 promoter region. Yeast strains transformed with the C/EBPα prey and normal *PxTreh*2 bait grew normally on the selective media that lacked Leucine (Leu) and contained AbA, while the transformant with the negative control did not grow under the same conditions (Figure 5D), indicating that there was an interaction between C/EBPα and the *PxTreh*2 promoter region.

## 4. Discussion

Insects are exposed to numerous environmental stressors, including chemical insecticides. In response, insects undergo varied behavioral, physiological, and genetic changes to adapt and survive under such conditions. Some insects overexpress detoxification enzymes, or their target sites undergo mutation. However, these changes demand energy. Trehalose is a nonreducing disaccharide present in the hemolymph of most insects. Numerous studies have demonstrated a (positive) correlation between intracellular trehalose levels and the ability of insects to survive under severe environmental stresses, such as starvation, osmotic and oxidative stresses, and extreme temperature. In this study, we found that, compared with the susceptible population, the trehalase activity and the expression of the *PxTreh* gene were significantly high in the resistant *P. xylostella* population, further demonstrating the high energy demand in adaptation to toxins. A similar phenomenon was observed in resistant and sensitive maize weevils [36].

Exogenous chemicals, such as 20E, methylpentadiene, and juvenile hormones, can increase the activity of trehalase enzymes in insects. An increase in the activity of the trehalase supplies the high energy needed for the survival of insects in stressful environments [37]. Numerous transcription factors, such as SREBP, FoxO, and CREB, regulate the metabolism of carbohydrates and lipids. Thus, transcription factors regulate blood sugar balance and the growth and development of insects, all related to stress resistance, reproduction, and aging [38,39]. However, the regulation of trehalase expression in insects before this study was unclear. We found that C/EBPα promotes the expression of the trehalase enzyme in *P. xylostella*. The expression of C/EBPα and trehalase was significantly higher in the Bt-resistant *P. xylostella* population relative to the sensitive population. Inhibition of the C/EBPα expression significantly decreased the expression of *PxTreh*1 and *PxTreh*2. Further analyses revealed that the co-expression of C/EBPα significantly increased the promoter activity of *PxTreh*1 and *PxTreh*2 in Hi5 cells. This study further confirmed that C/EBPα regulates metabolism and energy production in insects. C/EBP is a pleiotropic transcriptional activator of adipocyte genes and regulates glucose and lipid metabolism [40], which is consistent with our findings.

Cellular transcription factors regulate gene expression by binding to specific DNA sequences (*cis*-acting elements). In this study, the binding sites of C/EBPα and *PxTreh* promoter regions were identified using a Y1H assay. Unfortunately, self-activation was detected with the bait vector of the region from −487 to +402 of *PxTreh*1, and interaction between the C/EBPα and the *PxTreh*1 promoter region could not be confirmed by the Y1H system. Although the Y1H assay indicated that there was an interaction between C/EBPα and the *PxTreh*2 promoter, the dual luciferase reporter system showed the predicted binding site mutation had no effect on the transcription of C/EBPα (Figure S1 in Supplementary Materials). These findings showed the expression of trehalase in *P. xylostella* is a complex process, and further research is needed to reveal the precise molecular mechanism that underlies the regulation of this gene. In general, this study revealed that C/EBPα promotes the expression of trehalase in *P. xylostella*.

## 5. Conclusions

We found that trehalase gene expression and activity are significantly high in Bt-resistant *P. xylostella* compared with Bt-sensitive *P. xylostella* strains, suggesting that trehalase genes mediate the adaptation of the insect to environmental stressors, such as insecticides. Further analyses revealed that the expression of *PxTreh* is positively regulated by the C/EBPα transcription factor, which mediates the adaptation of *P. xylostella* to adverse stressors. Finally, the relationship between C/EBPα and *PxTreh*2 promoters was verified, which aids in elucidating the molecular process of the adaptability of *P. xylostella* to adversity.

## Figures and Tables

**Table 1:** Oligonucleotide primers used in this study.

Primer Name	Sequence (5′ to 3′)	Description
PxTreh1-F	CCGAGAAGGCATCAAGAAC	Promoter cloning
PxTreh1-R	GAGTCGTGGAAGATGCGC	
P(−1188/+402)	GGCTCGAGATCTGCGATCTAACTCGTTCGTTCGTTCGTG	Recombinant upstream
P(−610/+402)	GGCTCGAGATCTGCGATCTAACGTACGATACCGATGTAGG	
P(−545/+402)	GGCTCGAGATCTGCGATCTAACTGCTAACGCCATCAGTG	
P(−487/+402)	GGCTCGAGATCTGCGATCTAACTGTCGCAATTTATTTCCT	
P(−426/+402)	GGCTCGAGATCTGCGATCTAACCTAGATGGCGCTGCTAT	
Promoter-R	TTAGATCGCAGATCTCGAGCC	Recombinant downstream
*PxTreh*2-F	GTGAGTTTTCCCACCCAT	Promoter cloning
*PxTreh*2-R	CGAGACCGAAATTAGAAGGAA	
P(−1892/+196)	GGCTCGAGATCTGCGATCTAAGTAGTGAGTTTTCCCACCCAT	Recombinant upstream
P(−1452/+196)	GGCTCGAGATCTGCGATCTGGTTTATTATCGTCCTATCAATG	
P(−925/+196)	GGCTCGAGATCTGCGATCTGAGTGGAGCCAGTCCATG	
P(−315/+196)	GGCTCGAGATCTGCGATCTGTGACACTGCATTTGAGCG	
P(−254/+196)	GGCTCGAGATCTGCGATCTCATCTAGCCAACCTGGGC	
P(−203/+196)	GGCTCGAGATCTGCGATCTAACACGTAAGGGGACGC	
P(−141/+196)	GGCTCGAGATCTGCGATCTAACGACACAGATCTAAAC	
P(−90/+196)	GGCTCGAGATCTGCGATCTGGCAACACTGAAATCAAGGC	
Promoter-R	TTAGATCGCAGATCTCGAGCC	Recombinant downstream
C/EBPα-F	ATGGAGTCCCCACAGATGT	TF cloning
C/EBPα-R	AGCGCGTGGTCGCCGTGC	
C/EBPα-Y1H-F	ATGGAGTCCCCACAGATGT	pGADT7-PxC/EBPα
C/EBPα-Y1H-R	AGCGCGTGGTCGCCGTGC	
qC/EBPα-F	TCAAGAAGAATGGCGAAGAC	qPCR analysis
qC/EBPα-R	CAGGTCCAGGCTGATCTC	
qTreh1-F	TTCCACGACTCCAAGACCTT	
qTreh1-R	CAGCAGCACAGCGAAGTC	
qTreh2-F	ATGCTCTCCAACTTCCTCAAC	
qTreh2-R	GCGGCTGTGACCTCATAG	
EF1-F	GCCTCCCTACAGCGAATC	
EF1-R	CCTTGAACCAGGGCATCT	
RSP4-F	ATGGATGTTGTGTCGATTGAAAAGA	
RSP4-R	GAGTGATGCGGTGGATGGTGA	
dsC/EBPα-F	T7-TGGACGAGCTCAACGGCCAG	dsRNA synthesis
dsC/EBPα-R	T7-TGAGCAGAGGAGGCAGCAGC	

## Data Availability

The data presented in this study are available in the article and supplementary material.

## Supplementary Materials

The following supporting information can be downloaded at: https://www.mdpi.com/article/10.3390/insects13040340/s1, Figure S1: Effects of C/EBPα on the activity of wildtype and mutation of *PxTreh*2 (−203 to 196) promoters.
