# Supplementary material for: C/EBPα Regulates PxTreh1 and PxTreh2 Trehalase-Related Bt Resistance in Plutella xylostella (L.)
Source: Insects. 2022 Mar 30;13(4):340. doi: 10.3390/insects13040340 (PMC9024946; doi:10.3390/insects13040340)
Supplement: Supplementary file 1 [file insects-13-00340-s001.zip › insects-1635682-supplementary.pdf]

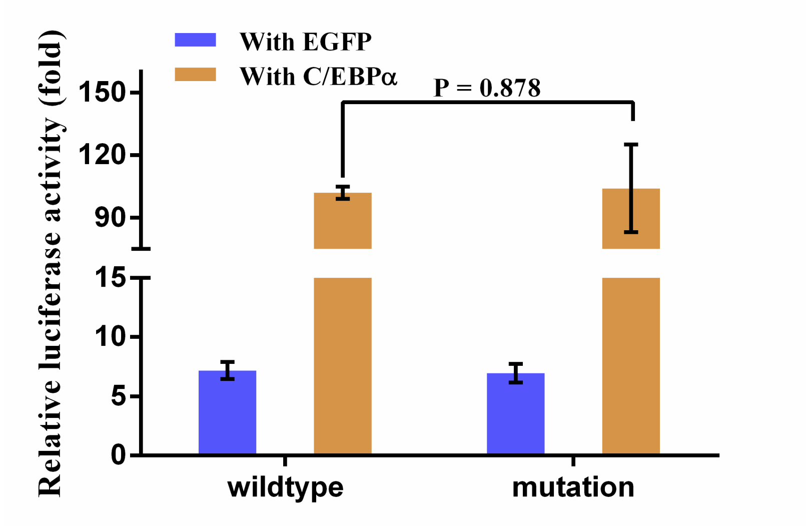

Figure S1. Effects of C/EBPα on the activity of wildtype and mutation of *PxTreh*2 (-203 to 196) promoters. The wildtype sequence "ATTAAAAA" in *Treh*2(-203/196) was changed into sequence " AAAGCTTA" in mutation group, the mutation did not affect the enhancement of C/EBPα ($P > 0.05$).
